# Polycentric vs monocentric urban structure contribution to national development

**DOI:** 10.1186/s44147-021-00011-1

**Published:** 2021-09-29

**Authors:** Ashraf Sami Mahmoud Abozeid, Tarek Abdellatif AboElatta

**Affiliations:** Department of Architecture, Faculty of Engineering, Cairo University, Giza, Egypt

**Keywords:** Independency, Satellite, Urban structure, Urban agglomeration, Urban diversity, Polycentricity, Concentricity, COVID-19

## Abstract

The debate about polycentricity and subordinacy has always been a critical topic that planners, economists, and socialists argued about for centuries. The idea of concentricity vs decentralization has affected all life metabolic activities. Urban structure has always been declared to be the key factor that affects life metabolism significantly. However, after the pandemic COVID-19, the planning strategies have changed dramatically. The main purpose is to investigate the most appropriate urbanization approach that achieves the best development results. The research methodology is to define and measure the fabric independency as an approach to estimate its self-sufficiency that enables it to stand in front of the pandemic challenges at different circumstances. The paper uses the fabric diversity index as a sensitive indicator of independency and polycentricity of the urban structure. The main conclusion for this paper is that independent polycentric urban agglomerations that are strongly linked achieve much better development results than subordinate cities depending on the main core city. The data used for the analysis are extracted from the Urban Atlas developed by the European Environmental Agency in addition to the UN-Habitat annual report. All calculations, analyses, and deductions are exclusively carried by the author.

## Introduction

### The research in brief

Development has always been the main critical topic that economists, socialists, and planners debated about for a long time ago. A lot of trials, theorems, and proposals have been developed to achieve the best development results since the early ages. The political argument and decision also varied according to the development desires and economic growth. However, theorists have reached by the end of the nineteenth century two main trends of civilization: the first is the very central concentric strategy that depends on the centrality of the decision to guarantee control of resources and actions, and the second is a decentralization strategy that depends on the democratic contribution of all parties in the governance.

Recently, major old cities have become overcrowded and saturated due to a massive accumulation of urbanization and evolutionary development that began a long time ago. The growing demand for housing and major activities, caused by the increase of population along the different eras, has encouraged planners to continue urban expansion. However, urban expansion was a debatable issue that urban planners, socialists, and economists have been arguing about for a long period of time.

To be more concise, many urban experts claim that the philosophy, strategy, and even the process of urbanization may influence the development rates to a great extent or can have an impact on economic growth. From this perspective, this paper investigates the most effective urbanization approach that can achieve the best development results on a major scale. Regionally, there are two main trends of the urbanization process that shape city planning: monocentric urbanization and polycentric urbanization.

The two main trends were reflected on the urbanization approaches as methods to achieve the highest development score to prove the validity of one at the expense of the other. Consequently, two main planning approaches appeared as urbanization strategies. *Monocentric planning* focuses on developing one main urban pole with related suburban areas in the vicinity of the main core.

The other is *polycentric planning* that focuses on developing multiurban poles that share nearly the same level of equity in most life aspects achieving what is known by urban equilibrium [1]. However, after the COVID-19 pandemic, different opinions and thoughts about city planning have aroused. Hamidi et al. (2020) [2] did not find a strong positive correlation between COVID-19 infection and mortality rates and density. The long-term economic shutdowns due to the COVID-19 pandemic have had very negative impacts on the urban economy. The consequence is complicated and occurs in different ways and on a wide range of scales (Krzysztofik et al. 2020) [3]. The traditional planning theories need to be revised and adapted to suit the new challenges that have appeared recently. In other words, a new meaning of development should be defined in the *new normal* that does not rely on economic growth only but a compromise between uniform production rate and sustaining residents’ and workers’ health across the urban fabric.

### Research argument

The main argument was about the best planning strategy that can achieve the best development scores. Surprisingly, compared with sprawling areas, they observed slightly lower virus-related mortality rates in high-density locations. A lot of researches and comparisons have been carried out to reach a conclusion to this debate.

However, in most cases, the gap in the literature was that the measurement approach was not accurate or biased that led to paradoxical results. This research tries to give an added value to the research about polycentricity topic by presenting a fair measurement methodology that takes into account all factors. *The main motivation* to carry out this research is to seek out a non-biased measurement approach and calibration method for polycentricity using reliable concrete data that are stable from one urban area to the other so that the comparison is a fair one at the end.

#### Research aim

The main aim of this research is to define a new approach for evaluating polycentricity so as to judge its impact upon development.

#### Research objectives

The main objective of this paper is to explore whether *monocentric urban structure* (represented in the core within subordinate cities planning model) or *polycentric urban structure* (represented in autonomous connected cities model) is the most appropriate and effective planning approach that contributes to development after the pandemic.

#### Research hypothesis

The hypothesis formulated for this research indicates that *independent autonomous urban agglomerations*, which represent the multicenter planning approach, have a greater contribution to development than *satellite cities*, which depends on the main core representing monocentric planning approach.

#### Monocentric and polycentric urban structure

The first model for the monocentric city model was generated by Alonso [4], then it was developed by Mills and Muth to include transportation, production, and housing. Fujita unified the previous models then in one framework. Ogawa and Fujita developed after that two-sector monocentric models of a one-dimensional city [5]. Fujita argued that the concentration of firms in one place increases the agglomeration zone, and consequently, the commuting distance for their workers on average increases and the wages as well [6–8]. Land rent around the agglomeration increases also. The rise in the cost of labor and land then discourages further firm agglomeration and encourages an opposite phenomenon to occur which is urban sprawl at the peripheries [9].

*Polycentricity* is a multiscalar concept that works at local, regional, and national levels [10]. The concept has been tackled as an approach to counter the core-periphery concept that used to be the major trend of urbanization. Some planners have stated that there is no single definition for polycentricity [11]. Two main major categories of definitions can be identified in the literature. First is *morphological* that focuses on population size, employment rate, land use combinations, etc. An area could be named a polycentric fabric if it contains two or more centers and population and employment are not concentrated in just one single center. The other definitions are oriented towards the *functional* approach [12]. It mainly emphasizes the activity exchange and metabolism of the fabric [13]. Klosterman and Mustard stated that “polycentricity can, in principle, refer to any clustering of human activity.” They summarized the characteristics of any polycentric urban area into two main features as follows:

- A group of connected distinct cities
- No obvious leading city

The first polycentric model was developed by Fujita and Ogawa [5–7]. The main hypothesis was that the benefit from cooperation between two firms is inversely proportional to the distance between them, i.e., when commuting costs are relatively high, this leads to the formation of multiple business cores and consequently achieves what is known by “multi-equilibria.” It is worth saying that the differences in the degree of production or transport cost lead to the variation in the size of agglomeration and, consequently, the spacing between industries. According to Fujita and Mori, the presence of multiple industries leads to the formation of a hierarchical city system.

Application of polycentricity and monocentricity can be reflected through the following two main urban structures that this paper discusses:

-First, independent linked urban agglomerations
- Second, connected satellite urban cities [14]

To understand the meaning of autonomous independent urban agglomeration, the definition of the term “urban agglomeration” should be clearly outlined. Table 1 shows the different definitions that have been associated with the urban agglomeration expression.

**Table 1:** Urban agglomeration definitions summary

Year	Basic opinions of urban agglomeration definition	Representative scholars
**1898**	Equivalent to town cluster	**Ebenezer Howard**
**1920**	Is an urban economic zone	**Beograd**
**1931**	Is a concentrated urban area	**Fawcett**
**1933**	Is a city cluster	**W. Christaller**
**1942**	Is an aggregate of cities	**R. Vining**
**1957**	Megalopolis (clusters of megacities)	**J. Gottman**
**1968**	Is urban expansion area	**T.Hager strand**
**1980**	Is a multieconomic center urban area	**J. Song**
**1980**	Equivalent to Metropolitan Inter-locking Region (MIR)	**Y. Zhou**
**1985**	Megalopolis and integrated core-peripherals	**D.A. Rondinelli**
**1985**	Comprehensive and integrated urban spatial organization	**J.B. Mcloughlin**
**1989**	A concentrated urban area with clear hierarchy	**L. Dong**
**1991**	Metropolitan belt	**N. Pyrgiotis**
**1992**	Integrated urban cluster	**S. Yao**
**2007**	A concentrated region of population and economy	**P. Ni**
**2015**	Highly integrated groups of cities	**C. Fang**
**2021**	A continuous urban spread constituting urban Spread constituting a town and its adjoining outgrowth	**Maitry U. Pate**

According to the above table, it is obvious that urban agglomeration has been defined through various approaches. The previous definitions could be briefly summarized into main four meanings. The first one defines urban agglomeration as an *urban area or cluster*, while the second states it as an *aggregate or concentrated urban area*. The third definition described it as an urban region that has a *diverse economic base* and products, and finally, the fourth definition portrayed that it is an urban area that forms a *metropolitan or megalopolis zone*. Using the evidence available, it is possible to deduce that not every urban agglomeration can be a candidate to be an autonomous zone, but there are some forms of urban areas that could be genuinely independent ones. This paper tries to investigate whether *independency* or *subordinacy* is the best strategy that should be tackled when pursuing urban expansion so that it could achieve the best development results. To be more focused, any urban expansion can undertake one of the following two urban forms:

- *Autonomous linked urban agglomerations* is an approach that counts on *intimately connected independent urban areas.*
- *Subordinate satellite-connected cities* is an approach that relies on developing *connected urban areas dependent on the main center.*

#### Satellite cities

The idea of satellite cities was influenced by the principles of the Garden City introduced by Ebenezer Howard. Oxford Dictionary of Architecture defines satellite towns as follows:

Towns that are self-contained and limited in size, built in the vicinity of a large town or city to house and employ those who would otherwise create a demand for expansion of the existing settlement, but dependent on the parent-city for population and major services.

Satellite cities of the twentieth century were influenced by the principles of the Garden City as introduced by Ebenezer Howard. Howard developed the idea of building garden cities that were planned limited in size and surrounded by a permanent belt of open space. The main goal for developing satellite cities was to alleviate the issue of overpopulation in the capital city without resulting in sprawl.

It is worth mentioning that satellite cities have been the most common trend throughout the last decades. Several urban planners and socialists argued that satellite towns and cities are a new approach that encounters a new implementation of the peripheries concept [15]. Satellite cities, on one hand, have proved to be a rapid strategy to develop an integrated urban area where inhabitants can find a better quality of life. On the other hand, it maintains the connection between the new urban agglomeration and the major city. This allows satellite cities to rely on the core city in many facilities and products requiring a large investment in infrastructure, which might be costly and takes a long time. At the same time, it solves a serious housing problem by providing new dwelling units at lower prices and more facilities compared to the limited ones existing in the capital city. It could be claimed that it contributes somehow to provide job opportunities at the service sector level but not at the level of the production sector. Some planners argue that job opportunities should not always be in the production sector in order to contribute to economic growth. They justify that the service sector acts as a wallet for gaining excess revenues from the residents. As a result, these revenues could be embedded in the production process. The speculative hypothesis is based on developing a well-integrated loop that securely carries out economic externalities from distribution centers to production centers and so on.

*In other words, a satellite city can be a potential to develop a new community with diverse amenities without being overburdened by the cost of creating unaffordable land use categories.*

On the other hand, adverse criticism has been directed to satellite cities. The main claim was that after these new urban communities were established, they proved to play an important role in shaping new urban settlements. Yet, they showed to have no significant impact on the national income. The reason behind this result is that the inhabitants rely on their job opportunities and life needs production upon the mother core city. In other words, the major core city is the place where the economic base, either industrial or agricultural, is found. It can be mentioned that it acts as a supplier and feeder to the associated satellite cities, which in turn act accordingly as channels for the distribution of these products and commodities to users and residents. Contemporary planners also argue that even relying on services as an economic portfolio is not effective. In most cases, large investment in infrastructure and transportation is required to transfer capital smoothly and quickly. Satellite cities are also criticized for not being true urban communities. That is, usually, these cities do not contain a wide cross-section of society either in terms of dwelling type or job categories. This is because inhabitants depend on satisfying certain life needs on commuting between the mother city and the subordinate ones.

It should be honestly stated that some of these satellite cities have proven to be quite self-sufficient. Yet, none of them has reached the level of autonomy or complete Independency [16]. This is because the concept upon which these cities were planned depends on the fact that they are dependent on the mother city. Consequently, they are not supposed to give an added value to urban income. In other words, the goal they were developed for was to solve a housing problem. Therefore, it is illogical to blame these cities for not contributing to the economic growth effectively or boosting development as well.

## Methods

The ESPON research program was developed to achieve a better understanding of spatial trends, problems, and opportunities on a European scale. Many versions were developed starting from the ESPON 1.1.1 project that focused on the role and potentials of urban areas as nodes in a polycentric development. The ESPON 1.4.3 project targeted to analyze Project 1.1.1 by delimitation of functional urban areas (FUAs) and analysis of polycentricity based on this approach. Both ESPON 1.1.1 and 1.4.3 sub-indexes were as follows: size index, location index, and connectivity index. However, both projects were criticized for the following reasons. First, the previous measurement approaches focused on defining polycentricity from a morphological issue concerning only size and territorial distribution. In addition, the need of finding a new measurement method comes from the inaccuracy in the measurement method by ESPON projects (Meijers 2008) [17]. Meijers stated that the results are based on too many FUAs. For example, in larger countries, the calculations for the flatness of the urban hierarchy and primacy are strongly influenced by the smaller FUAs. It is also worth saying that as used in ESPON 1.4.3, a fixed size threshold has its disadvantages. A city ranked 10th in one country could be an important one in a smaller country. Such a measure twice would deviate the results and distort the picture. Meijers then concluded that measurement of primacy should be calculated relative to a small fixed number of FUAs about *n* = 4 or 5 (Meijers, 2008) [17].

**Fig. 1:**
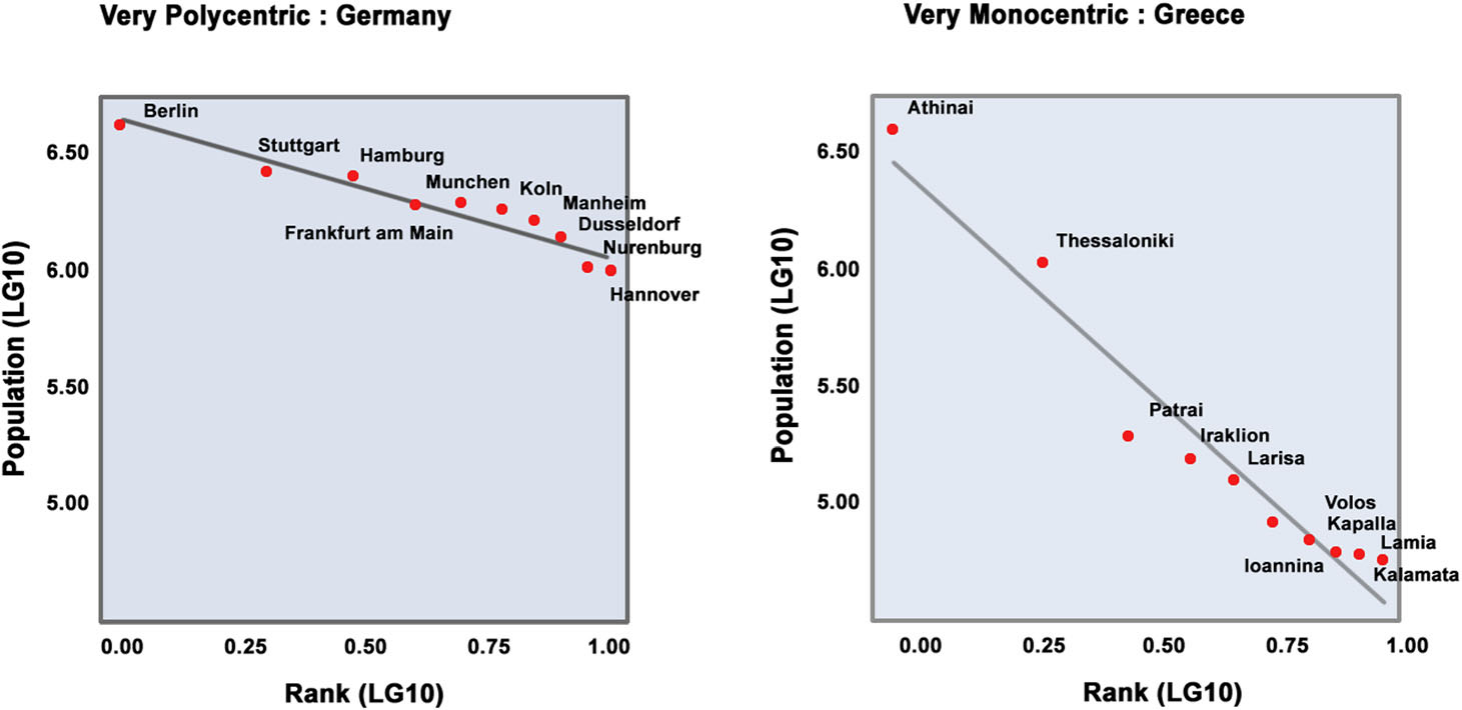
Polycentric and monocentric countries in ESPON 1.4.3

An example of rank size approach as a parameter of polycentricity index developed by ESPON 1.4.3 is presented in Fig. 1 that shows the rank size distribution of Germany and Greece. The figure shows Germany is a very polycentric country where cities are nearly equal in size. On the other side, Greece is a very monocentric country, the cities vary significantly in sizes and are composed of core and subordinate cities.

It was agreed above that the objectives of this research are to measure the level of independency and polycentricity for urban agglomerations and subordinate ones to know to what extent it impacts economic externality production and development. From this prospective, it is evident that a sensitive benchmark index should be defined whereas it could be a genuine indicator of independency and polycentricity. On the other hand, development scores should be formulated based on a reliable standard source that guarantees the accuracy of the results [18].

Independency is a two-dimension characteristic. The *morphological* dimension ensures that the urban area from the urban form is an identifiable agglomeration. Besides, it also verifies that it is detached and not an attached mass to another one. The *functional* dimension guarantees that the urban area’s metabolism works efficiently. Some scholars have used *density* as an indicator of the intensifying factor for the morphological approach. Others have used the *employment rate* and *dwelling occupancy* as indicators of population productivity performance for the functional approach. They justify that the high employment rate indicates that the urban area is well performing and quite well productive [19].

This paper contradicts this approach because the density can be in certain cases an accumulation of monoclass of inhabitants within a few variations in repetitive land use as well. This redundancy in the category of inhabitants and land use can cause either of two phenomena. The first phenomenon is a spillover effect where the surplus of one product causes a great loss in its price as the supply is greater than the demand. The second phenomenon is the competition that arises between similar products which may negatively affect the productivity rate overall.

It is also not preferred to use the employment rate or housing occupancy as indicators. This is because employment could be a standardized monotonic type of job that does not boost economic growth as desired, but fictitious hours of work that have no significant added value. Dwelling occupancy can be decisive, too. It might show that inhabitants always have a place to settle and live in, but in reality, they might not enjoy a good quality of life. Even relying on top-notch housing records is not even correct. Some people may possess multiple high-standard housing units though they do not exceed 5% of the community.

On the other hand, *diversity* can be a reliable indicator. The diversity of land use illustrates how this urban fabric has non-monotonic urban metabolism. In addition, it offers a wide range of possibilities for different activities, employment categories, and even inhabitants’ classes. Diversity is claimed to be a quite accurate indicator that considers the variability and differentiation between distinct categories. This helps to develop some relations between different analyzed categories, and the best appropriate combination between them as a whole.

In this paper, the diversity indices proportions are used as a trustworthy indicator of *Independency*. The paper suggests that the Independency of any urban agglomeration is a main indicator of self-sufficiency. Self-satisfaction can be measured using numerous methods. Yet, depending on activity rates and records could not be an accurate approach. This is because the type, nature, and frequency of activities vary from one place to another. In addition, a standardized criterion that ensures neutralization of other third-party political interference or any factors that may lead to the deviation of the results should be carefully considered. Comparing non-solid parameters, such as employment rate, illiteracy, and number of dwellings, is always decisive as well. On the other hand, development records are going to be extracted directly from *UN-Habitat Sustainable Development Goals Report* as a reliable standard source that contains accurate numbers and scores for the main development aspects as defined by the United Nations.

### Measurement of independency (polycentricity)

The fundamental concept upon which the research is based is the principle of primacy as a measurement approach for urban agglomeration ranking. On the national scale, a country ranking can be easily determined by measuring the primacy of the largest agglomeration to the rest of urban areas. The hypothesis denotes that if the dominant urban area shows higher urban primacy with respect to other urban areas, then it tends to be a core with dependent peripheral satellite cities (*a monocentric planning approach*)*.* On the other hand, if the dominant urban area shows relatively near rank-size compared to other urban areas, then this urban fabric is a network of Autonomous Independent Urban Agglomerations (*a polycentric planning approach*)*.*

#### Polycentricity index calculation

Calculation of urban primacy and polycentricity of a country is through the following:

- Calculation of the diversity index ***H***_**urbn**_ of the major four urban agglomerations within every country based on the land use data available (*The major four urban agglomerations selected are the largest functional urban areas in size and population within every country according to the European Union Statistical Agency, Eurostat, 2016. As Meijers(2008)* [17] *has stated that when measuring polycentricity, the largest urban areas should only be selected to guarantee the neutrality of the size factor upon the results. In addition, the location of cities is not taken into account as the goal is to measure the polycentricity of cities which is different from measuring the city networking effect).*
- Every country has, therefore, a dominant urban agglomeration identified by the largest diversity index value ***H***_**Major_Urbn**_ (usually the country capital), and other three major urban areas whose diversity index were also calculated.
- The standard deviation is calculated for the four diversity index values of the chosen cities for each country as ***σ***_**cntry**_.
- The mean is calculated for the four diversity index values of the chosen cities for each country as ***μ***_**cntry**_.

The magnetic effect of mutual attraction between the different centers is either multiplied or reduced using the coefficient of variation. The coefficient of variation for each country **CV**_**cntry**_ is calculated by dividing the country standard deviation by the country mean as shown in the following equation:

1 $$ {\mathbf{CV}}_{\mathbf{cntry}}={\boldsymbol{\sigma}}_{\mathbf{cntry}}/{\boldsymbol{\mu}}_{\mathbf{cntry}} $$

High values show significant variations between the diversity indices, while low values show that the diversity indices of the cities are nearly the same.

The dominant city area diversity index ***H***_**Major_Urbn**_ is multiplied by the inverse of the coefficient of variation for each country (**CV**_**cntry**_) to indicate the country’s polycentricity effectuation, impress, and degree (*P*/*I*) as shown in Eq. 2.

2 $$ \boldsymbol{P}\left(\boldsymbol{I}\right)={\boldsymbol{H}}_{\mathbf{Major}\_\mathbf{Urbn}}\times \left(1/{\mathbf{CV}}_{\mathbf{cntry}}\right) $$

High values indicate nearly high equal diversity indices among the different cities, and the country tends to be highly polycentric. Low values indicate low polycentric effect, and the country tends to be a monocentric one.

#### Urban diversity calculation

As mentioned before, urban diversity is a sensitive indicator of polarity and independency. Shannon entropy was chosen to be the method to measure urban fabric diversity using Eq. 3 [20]:

3 $$ \mathbf{H}=-\sum \limits_{i=1}^s{p}_{\mathbf{i}}\ \mathbf{\ln}\left({\boldsymbol{p}}_{\boldsymbol{i}}\right) $$

*H* = the Shannon diversity index value

*Pi* = the proportion of individuals found in the *i*th species

ln = the natural logarithm

*s* = the number of species in the community

By applying Eq. 3 to the case of urban areas, *Pi* is the proportion of the *i*th land use area to the total city functional urban area (F.U.A). *S* is the no land uses in the urban area. The methodology upon which the land uses that shape the urban agglomeration diversity index were chosen undergoes the following conditions:

1. Land use should be a category of any urban development (rural land use categories are not taken into account).
2. Sprawled urbanization was excluded from the research analysis. Since the research is oriented towards urbanization that influences development, it was decided to choose the main land uses that reflect the main flow of the labor force rather than any other land use that would have arisen under special circumstances. In addition, low-density scattered urban areas cannot be accurately identified. They could either be luxurious areas with entertaining green open areas or just poor informal buildings lying in the peripheries. In both cases, data about the nature of residents in these areas and their activities is always missing and decisive.
3. Diversity is claimed to be the most appropriate indicator of a balanced land use mix. High index values reflect fine coherent proportions of land use combination, while low values point out that the fabric is a coarse one. Accordingly, the following land uses were selected to represent a variety of urban societies:
  - *Continuous urban fabric* [C.F] (it is a fabric type where the urban surface is majorly covered by impermeable features, such as buildings, roads, and artificially surfaced areas.)
  - *Discontinuous high-density urban fabric* [D.D.F] (it is a fabric land use where the impermeable features, such as buildings, roads, and artificially surfaced areas, range from 50 to 80% land coverage.)
  - *Discontinuous medium-density urban fabric* [D.M.F] (it is a fabric land use where the impermeable features, such as buildings, roads, and artificially surfaced areas, range from 30 to 50% land coverage.)
  - *Industrial or commercial units and public facilities* [I/C.F]. This category is assigned for land units that are industrial or commercial use or public facilities.
  - *Railway network* [R.F].
  - *Urban green areas* [G.F].
  - The selected area represents the major countries in Europe. North countries are excluded from the comparison
  - as they have developed in different prosperous circumstances. The countries were chosen to represent Western, Eastern, and Southern Europe. All data for land use areas for the thirteen European cities was calculated using GIS shapefiles located in a project called “Urban Atlas” certified by the European Environment Agency an agency of the European Union [21]. Table 2 highlights the above-defined land use values for thirteen countries in Europe. It also shows the calculations for *Shannon diversity index* (***H***) for city urban agglomeration and *the polycentricity index* (***P***) for each country at the end. The criteria for selecting the countries is to have a comparison between countries with different backgrounds and situations. For example, Western countries represent the wealth and prosperous ones. South represents the Mediterranean diverse cultural ones. The Eastern European countries represent lower standard of living compared to Western countries.

**Table 2:** Urban fabric land use areas for the 13 selected European countries

Country	City	C.F*	D.D.F*	D.M.F*	G.F*	I/C.F*	R.F*	F.U.A*	(***H***)	CV	***P***(***I***)
**Germany**	Frankfurt	85.72	204.63	50.04	25.83	163.57	17.15	4300.38	0.45	0.122	**4.11**
	München	54.65	259.19	78.91	59.02	157.14	16.38	5195.2	0.44		
	Berlin	86.08	430.6	355.51	120	661.47	49.77	17,455.74	0.37		
	**Stuttgart**	**82.67**	**211.16**	**56.37**	**45.51**	**163.02**	**12.4**	**3650.82**	**0.5**		
**UK**	London	40.79	674.6	649.27	301.32	513.01	38.16	9094.34	0.7	0.156	**6.40**
	**Liverpool**	**16.24**	**129.12**	**30.86**	**41.86**	**68.97**	**3.33**	**645.49**	**1.0**		
	Manchester	13.99	187.37	114.12	64.16	148.11	6.62	1275.95	0.97		
	Birmingham	6.3	216.69	122.22	53.17	139.52	6.55	1597.08	0.84		
**France**	**Paris**	**226.41**	**563.06**	**344.4**	**207.21**	**526.11**	**53.38**	**12,068.57**	**0.55**	0.211	**2.61**
	Marseille	28.9	79.61	75.43	17.98	65.68	4.76	3173.53	0.34		
	Toulouse	30.83	214.13	107.1	11.84	110.16	4.91	4038.86	0.41		
	Lyon	46.45	214.66	87.41	28.18	130.48	12.96	3317.72	0.51		
**Netherlands**	Amsterdam	45.5	59.6	20.38	36.19	63.99	5.9	1172.48	0.64	0.106	**7.74**
	Rotterdam	36.13	50.29	21.21	30.98	53.33	6.6	708.66	0.82		
	Eindhoven	22.66	23.71	12.05	10.36	36.31	0.79	327.05	0.73		
	**Utrecht**	**13.5**	**29.59**	**18.19**	**13.46**	**28.26**	**1.87**	**389.64**	**0.787**		
**Belgium**	Antwerp	22.18	43.33	54.79	19.34	62.38	11.24	944.42	0.49	0.217	**3.56**
	Brugge	4.48	18.8	12.34	3.47	19.83	2.02	411.96	0.51		
	**Gent**	**7.33**	**33.25**	**47.8**	**8.9**	**48.57**	**5.08**	**539.49**	**0.77**		
	Brussels	31.06	54.78	107.51	41.25	87.51	9.7	1623.95	0.65		
**Italy**	**Naples**	**44.06**	**51.38**	**30.3**	**10.17**	**75.51**	**3.92**	**566.56**	**0.95**	0.345	**2.76**
	Milano	59.6	88.53	57.59	32.57	177.24	9.4	1344.09	0.84		
	Rome	59.2	135.29	116.08	41.81	168.06	10.58	3595.23	0.51		
	Torino	33.4	37.92	37.48	18.82	119.56	5.68	1879.3	0.47		
**Slovakia**	**Bratislava**	**18.51**	**64.47**	**18.21**	**7.45**	**72.98**	**5.83**	**2045.92**	**0.35**	0.141	**2.49**
	Presov	6.15	26.66	9.11	1.72	14.38	0.96	933.82	0.26		
	Zilina	13.13	20.47	2.51	1.52	12.68	1.93	813.79	0.27		
	Kosice	17.15	40.28	14.33	4.04	38.45	5.57	1774.73	0.28		
**Spain**	**Barcelona**	**75.35**	**56.43**	**51.31**	**20.34**	**128.5**	**7.71**	**1799.52**	**0.61**	0.395	**1.54**
	Sevilla	45.56	21.47	16.6	12.58	66.32	3.02	3079.51	0.24		
	Valencia	37.26	16.29	16.75	9.4	72.13	3.39	1447.66	0.393		
	Madrid	86.98	118.18	89.78	100.09	251.1	14.76	8022.04	0.34		
**Greece**	**Athena**	**108.27**	**125.28**	**5.07**	**27.95**	**131.47**	**2.98**	**3040.45**	**0.45**	0.462	**0.98**
	Thessaloniki	31.33	40.56	10.77	3.32	51.84	0.85	1425.82	0.36		
	Larisa	10.57	17.06	6.23	1.15	23.29	1.71	1555.69	0.18		
	Ioannina	4.5	16.14	7.86	1.25	22.21	0.02	1326.32	0.18		
**Hungary**	**Budapest**	**148.71**	**253.37**	**60.44**	**37.86**	**138.89**	**12.14**	**2522.5**	**0.74**	0.472	**1.57**
	Debrecen	18.77	56.78	16.04	4.56	43.47	3.63	1675.52	0.33		
	Nyiregyhaza	22.4	71.06	17.71	1.19	29.15	3.01	1436.61	0.37		
	Kecskemet	12.61	42.85	13.2	4.48	34.07	2.96	1482.24	0.3		
**Poland**	Warsaw	281.41	216.91	19.91	38.3	158.79	16.18	5201.72	0.47	0.295	**2.24**
	Poznan	61.11	115.87	16.54	35.31	98.04	9.13	3716.15	0.35		
	**Katowice**	**82.24**	**237.96**	**14.79**	**46.65**	**177.04**	**28.75**	**2635.79**	**0.66**		
	Lodz	36.82	149.19	24.19	13.37	82.23	5.89	2856.74	0.39		
**Czech**	Prague	106.34	268.72	62.45	79.7	191.48	23.18	6969.21	0.40	0.232	**1.85**
	Brno	23.59	94.38	52.32	8	74.64	6.59	3299.23	0.32		
	**Ostrava**	**71.99**	**156.9**	**54.59**	**35.16**	**108.28**	**14.16**	**3886.76**	**0.43**		
	Plzen	17.63	89.28	20.62	3.66	49.15	5.82	3103.01	0.25		
**Portugal**	Lisbon	90.02	88.4	39.18	26.39	97.34	4.07	1435.97	0.72	0.526	**1.73**
	**Porto**	**48.32**	**61.42**	**25.59**	**14.29**	**46.21**	**1.39**	**562.74**	**0.91**		
	Braga	6.96	20.98	18.23	2.54	13.4	0.28	493.44	0.45		
	Faro	5.13	4.47	3.14	0.47	9.71	0.44	481.91	0.22		

*All areas are calculated in square kilometers

*F.U.A = *functional urban area* which is an area that includes the city limits in addition to the commuting zone associated within it. It is calculated by Eurostat, the statistical agency of the European Union (EU) in cooperation with Urban Audit, 2016

*This approach for calculating polycentricity is a sort of measuring *land use urban equilibrium* for the main four cities within a European country

*Cities in bold are the major urban cities that scored the highest diversity index within every country

### Development Index formulation

The second element in the comparison is development. In order to accurately measure development, the UN-Habitat defined a new comprehensive meaning of development that is not only limited to economic growth but extends to take into account all elements and factors that sustain growth. The new development term is claimed to be achieved through certain goals they declared as a measurement approach for development in general. In fact, a lot of criticism was directed to the UN-Habitat definition for development as it included many parameters and aspects while many urban planners and economists define development only as “the economic growth that pursuit positive change for the society.” The new definition goes beyond the limited understanding of city development to be a comprehensive developed resilient one in the “new normal.” Table 3 highlights the values of every goal for each of the selected thirteen countries by using a color code. The code classifies the values into four main categories of goal fulfillment. Green indicates goal achievement, yellow challenges remain while orange shows significant challenges, and at the end, red indicates major challenges [22]. The seventeen goals are as follows: *no poverty*, *zero hunger*, *good health and well-being*, *quality education*, *gender equality*, *clean water and sanitation*, *affordable and clean energy*, *decent work and economic growth industry*, *innovation and infrastructure*, *reduced inequality*, *sustainable cities and communities*, *responsible consumption and production*, *climate action*, *life below water*, *life on land*, *peace and just strong institutions*, *and partnerships to achieve the goal.*

**Table 3:** Development index developed by UN-Habitat extracted from UN-Habitat SDGs annual report 2019

Country	ID	SDG Score (0-100)	G1	G2	G3	G4	G5	G6	G7	G8	G9	G10	G11	G12	G13	G14	G15	G16	G17
**Germany**	DEU	**75.35**	Y	O	Y	Y	Y	Y	O	G	G	Y	Y	R	O	R	O	O	Y
**France**	FRA	**74.68**	G	O	Y	O	Y	Y	Y	O	G	Y	Y	O	O	O	R	O	Y
**Netherlands**	NLD	**71.81**	G	R	Y	Y	Y	Y	O	Y	G	Y	Y	R	O	R	R	O	R
**Czech Republic**	CZE	**71.77**	G	R	O	O	O	Y	O	Y	Y	Y	O	O	Y	O	O	Y	O
**Belgium**	BEL	**70.29**	Y	R	Y	O	O	Y	O	Y	G	Y	Y	O	O	O	O	O	O
**UK**	GBR	**70.22**	Y	O	Y	Y	Y	Y	O	Y	G	O	Y	R	R	R	R	Y	O
**Spain**	ESP	**66.76**	O	R	Y	O	Y	Y	O	R	O	O	O	O	O	R	R	Y	O
**Portugal**	PRT	**66.23**	Y	O	O	Y	O	O	O	Y	R	O	O	R	O	R	R	Y	O
**Poland**	POL	**66.15**	Y	O	Y	Y	O	Y	O	Y	O	O	O	O	R	R	O	O	Y
**Italy**	ITA	**65.29**	O	O	Y	O	O	Y	O	R	O	O	O	R	R	R	O	O	O
**Slovak Republic**	SVK	**65.23**	Y	O	Y	O	O	Y	O	Y	O	Y	O	O	R	O	O	Y	O
**Hungary**	HUN	**65.12**	Y	O	O	O	O	O	O	Y	O	Y	O	O	O	O	O	O	O
**Greece**	GRC	**58.9**	O	O	O	O	R	Y	O	R	R	O	O	R	R	O	O	O	Y

*Y* yellow, *G* green, *O* orange, *R* red

By the aid of the previously mentioned seventeen goals, a development index was developed as a mean value to the seventeen scores indicating the degree of development progress for each country as shown in Table 3.

## Results

**Fig. 2:**
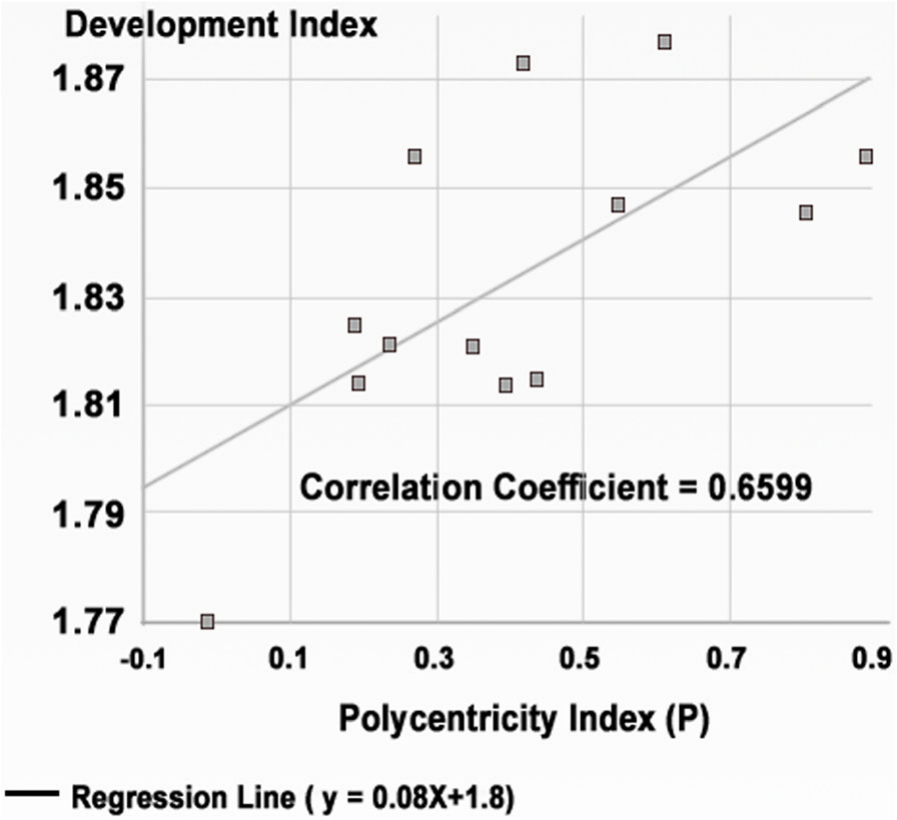
The relationship between the polycentricity index and the development index for selected 13 European countries

A relationship between the developed polycentricity/independency index and the calculated development index by the UN-Habitat was established as shown in Table 4, in order to investigate to what extent independency or concentricity can influence the development process (Fig. 2). The development values were standardized using the logarithmic normalization approach to conserve the analysis from any deviation or error.

**Table 4:** Polycentricity index values and normalized development index values for 13 European countries

Polycentricity index log	Development index log
0.614	1.877
0.806	1.846
0.417	1.873
0.889	1.856
0.551	1.847
0.440	1.815
0.396	1.814
0.189	1.825
− 0.011	1.770
0.195	1.814
0.350	1.821
0.268	1.856
0.238	1.821

It is clear from the chart shown in Fig. 1 that countries with a high polycentricity or independency index tend to show high values of development index. The relationship is considerably uniform and directly proportional. Concentric-based countries show lower development index values than those based on the polycentric approach. The Pearson correlation coefficient *R* = 0.6599 means that a quite strong relationship was found between macro urban fabric polycentricity and achieving high values of development. The analysis obviously explains that countries that host independent urban agglomerations record higher values of development. However, countries that host a primary core city with dependent satellite cities show lower values of development.

## Discussion

The paper has demonstrated the two main approaches of urbanization whether a concentric one depending on a core and peripheries or multicenter nodes that are linked and connected together. The paper investigates the best urban practice for city planning. A resilient urban center that can cope alone with the different circumstances has been declared as the best urban model. Consequently, independency has been chosen as the best measurement approach for center definition and elaboration. In other words, not any urban agglomeration can act as a long-lasting center, but it should be self-sufficient first. In fact, the diversity index for land use as an indicator of independency is claimed to be a sensitive measuring approach to macro urban structure polycentricity as well, i.e., diversity within unity reflects a fine combination of residential categories. *Continuous dense in the CBD, discontinuous dense at the outer ring, and even medium dense at the peripheries give the opportunity to integrate green areas and other land use within the residential fabric combating informal scattered sprawl at the end. It also provides a quite good mix of serving and production employment through commercial and industrial land use* [22]. This plays an important role in reducing commuting across the city and save time and money in the end. It should be honestly stated that independency generated from intra-fabric interactions is obviously reflected on the inter-fabric metabolism. It is also worth saying that diversity inherits the allocation of land uses in its perfect location to maximize the benefits from the activities carried out within each land use, i.e., some land uses if located in inappropriate positions might minimize its benefit or has a negative impact. For example, continuous urban fabric that is mainly compacted areas has its greater impact when centered in the city as it acts as a CBD for the urban area. Also, medium-dense fabric works efficiently at the peripheries as it plays an important role in combating sprawl by creating porous fabric at the outskirts instead of informal scattered areas. The variation in the social distances across the fabric promotes new activities and experience (Yunda and Jiao 2019; Abusaada and Elshater 2020) [23]. For instance, we can emphasize opportunities for meditation can be developed besides guiding people towards engaging in multiple areas of interest.

Epidemics and pandemics played an important role in urban history. The creation of parks, promenades, and public squares in European cities, for example, were early trials to provide safer urban spaces. Perhaps the largest impact was the rise of the public health movement in the nineteenth and twentieth centuries. Public health urban initiatives were attempts to develop open spaces in the cities as porous areas that consume the exhaust resulted from activities performed within the fabric areas [24]. This potential was not taken into account carefully before the pandemic. It was considered a sort of general public health safety precautions. Yet, it proved to be a crucial land use fabric that combats the pandemic implications and minimize its effect.

Results have broadly demonstrated that polycentricity when properly applied in its true and deepest sense plays an important role in boosting the economic growth and development at the end. Some economists and planners had chiefly criticized the idea of decentralization and independency. They still advocate the traditional model of concentric planning and subordinate urban expansion as the most successful model. To judge whether polycentric urbanization is achieving satisfactory outcomes in the development process, it should be explicitly stated that polycentricity works efficiently when applying the concept of *synergy*. Synergy means that the formation of a combination is more effective than the simple aggregate of its parts. It also requires that every part should be independent, self-sufficient so that when it collaborates with one another, the assembly is a neo complex profitable added value. On the other hand, satellite cities are based on complementing needs. Complementarity inherits the dependency of one part on the other, i.e., it could just be a simple exchange of raw materials to form an ordinary product. In addition, relying on one urban agglomeration upon the other could be understood in the traditional circumstances. However, in a pandemic such as the COVID-19, dependency of an urban area could be catastrophic. To be clearer, when an urban area is infected, most of the production and service activities stop as a result of the complete shutdown. Here comes the best benefit of polycentricity in combating the spread of the pandemic. As mentioned, polycentricity depends upon urban independency [24]. Therefore, when an urban area is infected, it could be isolated from the others until it recovers without infecting any other areas. At the same time, being self-sufficient promotes the infected zone to sustain alone and recover as a result of economies externalities it generated in the past with no need of major aid from other urban areas. On the contrary, in the monocentric model, all urban areas depend upon the main core in the essential needs. If the core is infected, a paralysis will affect all other urban areas dependent on the core, and the whole life activities in the metropolitan area will stop. Overall, while the link between COVID-19 prevalence and urban design characteristics has created many debates in the media and the public, the existing literature does not specify in much detail how different design measures such as connectivity, block size, land use mix, and polycentricity influence the infection and mortality rate of COVID-19 and the capacity of cities to respond to the pandemic. However, according to the early findings, planners are recommended to keep advocating compact forms of urban development rather than sprawling ones because various other benefits of compact urban development are demonstrated in the literature [25] (Connolly et al., 2020b; Hamidi et al., 2020; Sharifi, 2019a, b).

The other foremost debate was that centralization leads to economic externality accumulation while decentralization leads to dispersion and fragmentation of investments. However, this paper concluded a major expansion of this argument. In a nutshell, it could be claimed that an independent urban agglomeration which can stand alone is the only candidate nominated to contribute to the polycentricity concept that achieves high development records. Contrarily, any other dependent subordinate agglomerations or satellite towns (whatever their sizes or population) may deviate the results and fail the whole idea of polycentricity in an oppressive approach or measurement method [9].

## Conclusions

To summarize, polarity is the key factor of achieving development through attracting investments. Attraction effect means finding *positive relations* between the different inputs not just blind accumulation that could have negative or repulsive effects. Concentric urban structure model is not benefitable as it inherits cooperation (*a sort of dependent neutral horizontal complementarity*). Polycentric urban structure model inherits collaboration between different users. At the beginning of polycentricity, a sort of independent vertical complementarity occurs to form a product (*mass production phase*). The ultimacy of polycentricity happens when a sort of synergic complementarity occurs between the independent actors to form a new product each time they combine (*innovative phase*). In a nutshell, collaboration is always needed to achieve development not cooperation [26]. This is because collaboration is an independent driven process while cooperation is a dependent one. *Saturation* is a critical case in polycentric planning because synergic collaboration process is a complex one that has a long-term effect reflected on the quality of life. In addition, if a center reaches the saturation phase, it automatically inherits the monotonical accumulation of investments and actors. It then loses its polarity and consequently its magnetic effect of attraction between the different actors becomes weaker. On the other hand, vertical collaboration is a pillar for fast complementarity, new market openings, economic boosting reflected on GDP per capita. In all cases, a balance between synergic and vertical collaboration is always needed to avoid market saturation and formation of repulsive poles instead of attractive ones. The desired urban equilibrium can achieve the best development scores as in the Germany example.

It is also considered that in the future, polycentric planning should take into account the study of main land use composition that achieve the best results of independency and productivity. In this study, the land uses were unified across the different countries to guarantee the accuracy of measurement. However, the composition of other different uses can give more benefitable and accurate results than the used compositions. The used land uses are general ones. A quite detailed land use could be a more precise indicator and representative of the metabolic interactions between the different land uses and consequently the impact on development.

## Data Availability

Data generated or analyzed during this study are included in this published article [and its supplementary additional information files]. The datasets generated and/or analyzed during the current study are available in the following link: *https://www.eea.europa.eu/data-and-maps/data/urban-atlas*.

## Abbreviations

C.F: Continuous urban fabric
D.D.F: Discontinuous high-density urban
D.M.F: Discontinuous medium-density urban fabric
I/C.F: Industrial or commercial units and public facilities
R.F: Railway network
G.F: Urban green areas
F.U.A: Functional urban area
CV: Coefficient of variation
P: Polycentricity index
